# Increased levels of active c-Src distinguish invasive from *in situ *lobular lesions

**DOI:** 10.1186/bcr2332

**Published:** 2009-07-07

**Authors:** Donghui Zou, Han-Seung Yoon, Ahmad Anjomshoaa, David Perez, Ryuji Fukuzawa, Parry Guilford, Bostjan Humar

**Affiliations:** 1 Cancer Genetics Laboratory, Biochemistry Department, University of Otago, 710 Cumberland St, Dunedin 9054, Aotearoa New Zealand; 2 Pathology Department, University of Otago, 201 Great King St, Dunedin 9016, Aotearoa New Zealand; 3 Oncology Department, University of Otago, Dunedin Hospital, 201 Great King St, Dunedin 9016, Aotearoa New Zealand

## Abstract

**Introduction:**

Mounting molecular evidence suggests that invasive lobular carcinoma (ILC) is developing from *in situ *lesions, atypical lobular hyperplasia (ALH), and lobular carcinoma *in situ *(LCIS). However, little is known about the mechanisms promoting the progression of lobular breast cancer (LBC) to invasive disease. Here, we investigated whether c-Src kinase, an established inducer of invasive states, contributes to the progression from ALH/LCIS to ILC.

**Methods:**

Immunochemistry for c-Src and other cancer-related molecules was performed on archived tissue specimens from 57 LBC patients. Relative c-Src activity was estimated by comparing fluorescence intensity of ILC with that of adjacent ALH/LCIS and nonneoplastic epithelia after staining with an antibody against active c-Src. Expression of active c-Src was correlated with markers of invasion and malignancy and with relapse among LBC patients.

**Results:**

Levels of activated c-Src were increased in ILC relative to ALH/LCIS (1.63-fold ± 0.24 SD) and nonneoplastic epithelia (1.47 ± 0.18 SD). Increased c-Src levels correlated with the activation of c-Src downstream targets (Fak, Stat-3) and the expression of mesenchymal markers. ILC cells with activated c-Src co-expressed metastatic markers (Opn, Cxcr4) and included cells positive for the cancer stem cell marker Aldh1. A tendency for high c-Src levels (*P *= 0.072) was observed among the seven LBC patients with relapsed disease.

**Conclusions:**

Our data indicate elevated c-Src activity in ILC relative to noninvasive neoplastic tissue. The associated molecular changes suggest that c-Src promotes LBC invasiveness by inducing an epithelial-mesenchymal transition. Therefore, c-Src antagonists might counteract the acquisition of invasiveness during LBC progression. Inhibition of c-Src may also affect ILC cells thought to have a high metastatic potential and to be capable of initiating/maintaining tumor growth. Together with the possible association between high c-Src levels and disease recurrence, our findings encourage the evaluation of c-Src antagonists for the treatment of LBC.

## Introduction

Antagonists of the kinase c-Src are gaining increased attention as chemotherapeutic agents in breast cancer. Both *in vitro *studies and transgenic models suggest a central role or even a requirement for c-Src during the development and progression of breast disease (reviewed in [1-3]). Importantly, c-Src activity is elevated in human breast cancer tissue relative to adjacent epithelium, and increased activity has been associated with a worse outcome [4-6]. The major potential of c-Src inhibitors is that they also may be active against triple-negative and otherwise resistant breast cancer, for which existing therapy is inefficient [2,3]. However, these data are based largely on the major breast cancer histotype, ductal carcinoma. Whether c-Src also has a role in lobular breast carcinoma (LBC, which includes some of the triple-negative tumors) remains to be shown. This is a considerable gap in knowledge, because the clinical management is more challenging for LBC compared with ductal disease, and the increase in LBC incidence is disproportionately high relative to other breast cancer histotypes [7]. Therefore, new chemotherapeutic strategies are particularly relevant for LBC.

How exactly c-Src promotes breast cancer is not clear but may involve an array of cellular processes including proliferation, motility, invasion, survival, and angiogenesis [8]. Increasing evidence from breast and other cancers, however, suggests that a key feature of c-Src is to drive adhesive and motility changes crucial for invasion and metastasis [3,9]. We have studied very early stages of diffuse gastric cancer and observed that c-Src activity is induced when cancer cells undergo an epithelial-mesenchymal transition (EMT) to invade beyond the gastric mucosa [10].

Similar to diffuse gastric cancer, LBC is characterized by a discohesive growth pattern due to downregulation of the cell-cell adhesion molecule E-cadherin [11]. Indeed, germline mutation of the E-cadherin gene (*CDH1*) predisposes to both diffuse gastric cancer and LBC [12,13]. Given this common etiology, the parallels between diffuse gastric cancer and LBC may extend beyond E-cadherin and include the events associated with progression to invasive disease.

Although no consensus has been established, molecular evidence strongly suggests that invasive lobular carcinoma (ILC) develops from lobular *in situ *lesions: atypical lobular hyperplasia (ALH) and lobular carcinoma *in situ *(LCIS) [14]. Thus, lobular *in situ *lesions appear not to be merely risk markers, but rather true, albeit nonobligate precursors of ILC.

To this end, we reasoned whether the progression from LCIS to ILC may require an increase in c-Src activity and a concomitant dedifferentiation of epithelial morphology. We thus assessed c-Src expression in a series of archived LBC samples and correlated its activity with cellular and clinical parameters to determine the role of the kinase in the progression of human LBC.

## Materials and methods

### Patients

Formalin-fixed paraffin embedded (FFPE) tissue was retrospectively obtained from 57 patients (age 42 to 97 years; average, 65.5 years) who had undergone surgery for lobular disease at the Dunedin Public Hospital (Dunedin, New Zealand). The diagnosis was confirmed by an experienced pathologist (H-S Y) on hematoxylin and eosin-stained sections. Paraffin blocks were selected based on the simultaneous presence of ILC and LCIS/ALH or ILC and nonneoplastic epithelium, respectively. The patients' clinicopathologic characteristics were retrieved from the Oncology Unit (Dunedin Hospital), the Pathology Department (University of Otago), and from local general practitioners (GPs). Patients received 5-fluorouracil as standard therapy. None of the patients was given hormone therapy. Ethical approval for the study was given by the Lower South Regional Ethics Committee of New Zealand. Under this approval (for the collection of archived tissue), the Ethics Committee deemed the request for consent as unnecessarily intrusive for cured patients and the relatives of those deceased. Specific informed consent was thus not required for this study, consistent with the prevailing ethical consensus in New Zealand.

### Immunochemistry

Immunohistochemistry and immunofluorescence were performed on 4-μm paraffin sections boiled in citrate buffer (pH 6). Antibodies against active c-Src (clone 28, dilution 1:600, order nr. AH00551) and phospho-(P)-Fak (against pY861-Fak, 1:50, 44-626G) were from Biosource (Camarillo, CA, USA). Stat-3 (1:1,000, 9139) and P-Stat-3 (1:100, 9131) were from Cell Signaling (Danvers, MA, USA). c-Src (1:1,000, sc-8056), Fak (1:1,000, sc-932), and rabbit CK18 (1:100, sc-101727) were from Santa Cruz Biotechnology (Santa Cruz, CA, USA). CD10 (1:100, NCL-CD10-270), Muc-1 (1:100, NCL-MUC-1), CK5 (1:200, NCL-CK5), CK14 (1:20, NCL-LL002), mouse CK18 (1:40, NCL-CK18), estrogen receptor (1:80, NCL-ER-6F), and progesterone receptor (1:200, NCL-PCR-312) were from Novocastra (Benton, UK). Vimentin (1:400, M0725) and N-cadherin (1:50, M3613) were from Dako (Glostrup, Denmark). Additional antibodies were β-casein (1:400, ab6408, Abcam, Cambridge, UK), Cxcr-4 (1;50, 35–8800, Zymed Laboratories, San Francisco, CA, USA), osteopontin (1:100, 499265, Calbiochem, San Diego, CA, USA), and Aldh1 (1:150, EP1932Y, Epitomics, Burlingame, CA, USA). Primary detection was performed as described [10]. Omission of primary or secondary antibodies or both was performed for specificity control. Where available, tissue with a known expression pattern was used as positive control (for example, samples of invasive colorectal and diffuse gastric cancer for c-Src). Additionally, the state-independent expression pattern (antibody against total protein) was examined as a control for state-specific antibodies.

### c-Src activity

Src activity was estimated by measuring the intensity of immunofluorescence after staining with the clone 28 antibody. Fluorescence images were selected that contained either ILC and ALH/LCIS components, or ILC and nonneoplastic epithelium, or all three components. For each component, an area of homogeneous composition was defined, and average fluorescence intensity was measured by using ImageJ software [15]. In the absence of homogeneous areas (for example, ILC), individual cells were encircled in a number to equal the total area of neighboring components (for example, LCIS or epithelium), and the cells' average fluorescence intensity was measured. The ratio between the intensity of the different components within the same image was used as a measure for the relative activity of c-Src. Alternatively, 250 to 1,000 invasive cancer cells were counted, and the proportion of cells with strong c-Src activity was determined.

## Results

### E-cadherin and differentiation status of investigated LBC samples

**Figure 1:**
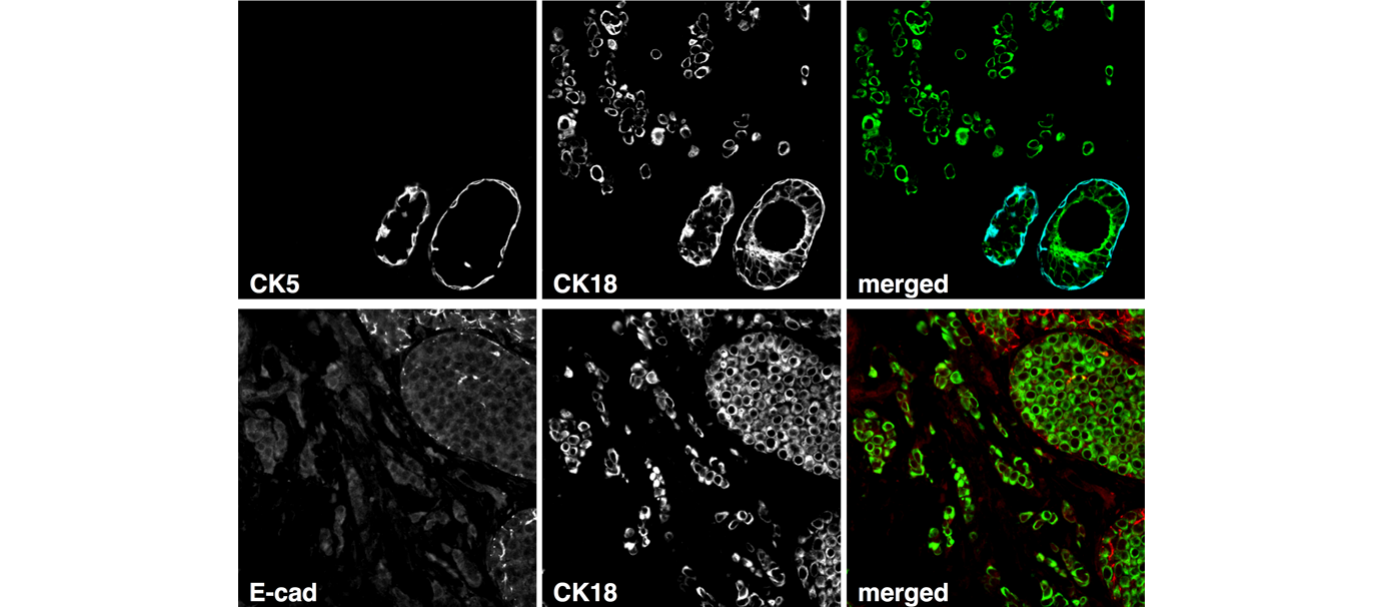
Differentiation of lobular breast cancer (LBC) cells. Upper panels: Nonneoplastic epithelium surrounded by invasive LBC cells. CK5 (blue) and CK18 (green) were detected with sequential staining, as both primary antibodies are from mouse. The basal cells (CK5) therefore stained with both secondary antibodies in this case. Lower panel: Invasive cancer cells and adjacent lobular carcinoma *in situ *(LCIS) stained for E-cadherin (red) and CK18 (green).

**Figure 2:**
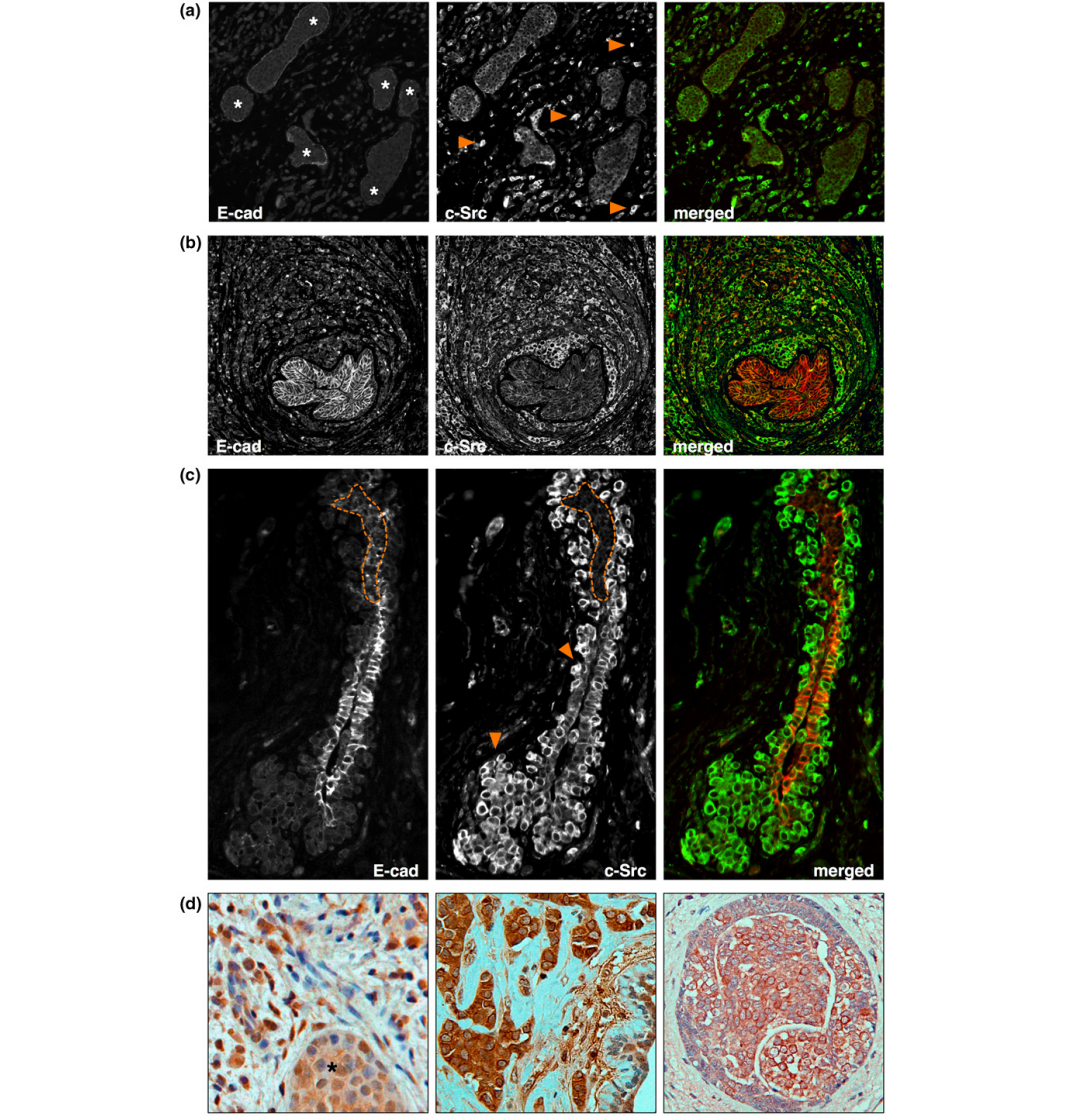
Elevated c-Src activity in invasive lobular breast cancer (LBC) cells. **(a) **atypical lobular hyperplasia (ALH) and lobular carcinoma *in situ *(LCIS) surrounded by invasive carcinoma cells. *In situ *lesions are marked with asterisks. Arrows point to examples of invasive cells with strong c-Src activity. **(b) **Nonneoplastic epithelium (membranous E-cadherin) surrounded by invasive carcinoma cells with active c-Src. **(c) **Nonneoplastic (E-cadherin) and neoplastic *in situ *components (dotted line) surrounded by and interspersed by invasive cells. **(d) **Immunohistochemical examples of an invasive lobular carcinoma (ILC) with moderately increased c-Src activity (left, LCIS marked with asterisk), an ILC with strong c-Src activity (middle), and a LCIS with low c-Src activity (right). To assess the extent of immunohistochemical c-Src staining, H-scores were determined to be 155 for ILC and 53 for LCIS (left), 291 for ILC (middle), and 51 for LCIS (right). An antibody against total c-Src was used as control.

Immunochemistry demonstrated downregulation of E-cadherin in all LBC samples available for this study (Figures 1 and 2). Staining with breast-lineage markers (cytokeratin 5 and cytokeratin 14 for basal cells, CD10 for myoepithelial cells, cytokeratin 18 and MUC1 for luminal cells, β-casein for alveolar cells) indicated that the vast majority (more than 90%) of LBC cells differentiated along the luminal epithelial lineage (Figure 1).

### c-Src activity is increased in ILC compared with LCIS and nonneoplastic mammary epithelium

**Figure 3:**
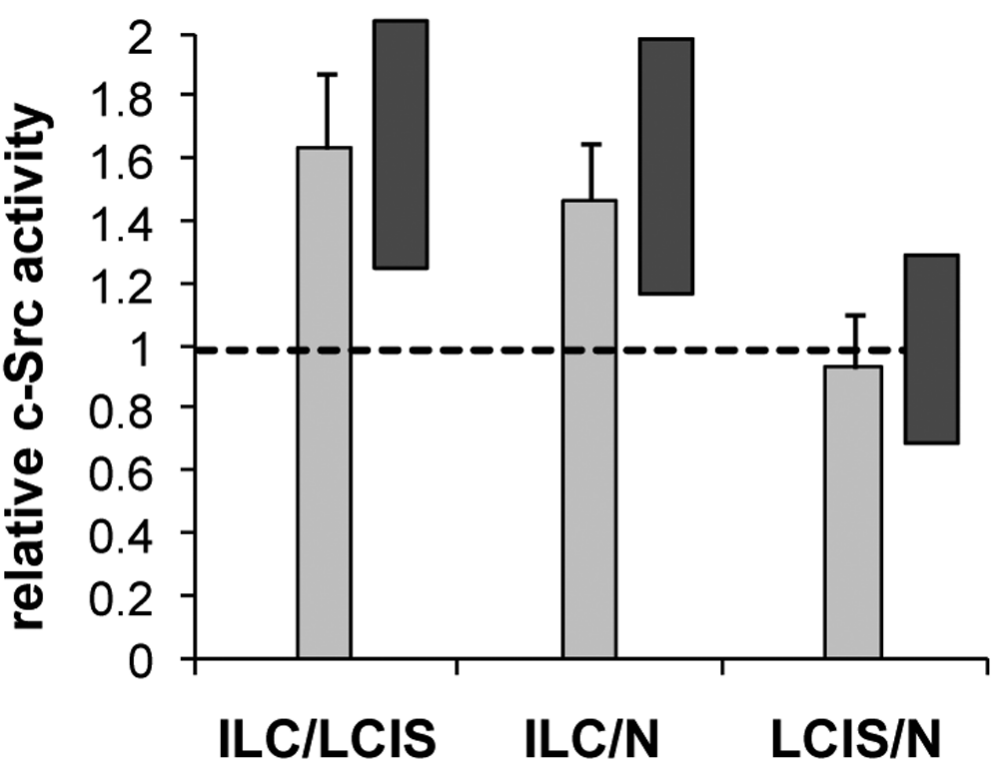
Relative c-Src activity in lobular breast cancer (LBC) samples. Relative fluorescence intensity in matched pairs of invasive lobular carcinoma (ILC) and lobular carcinoma *in situ *(LCIS), of ILC and nonneoplastic epithelium (N), and of LCIS and nonneoplastic epithelium. LCIS includes atypical lobular hyperplasia (ALH). Bars represent the average intensity ratio between indicated components from 57 LBC samples, and error bars represent the corresponding SD. The dark side bars indicate the data range.

To assess the activity of c-Src in ILC relative to *in situ *lesions and nonneoplastic epithelium, immunochemistry with the clone 28 antibody was performed on FFPE sections from 57 LBC patients. In all cases, expression of active c-Src was membranous/cytoplasmic on immunofluorescence. In the majority of samples, nonneoplastic epithelium and *in situ *lesions displayed moderate expression of activated c-Src. In contrast, c-Src activity appeared increased on visual inspection in invasive carcinoma cells relative to adjacent ALH/LCIS or epithelium (Figure 2). To obtain an objective measure for the relative c-Src activity, fluorescence intensity was determined across ILC, LCIS, and epithelial components, and intensity ratios were calculated for each sample separately. Averaged across all samples, fluorescence intensity was 1.63 (± 0.24 SD) times higher in ILC relative to LCIS, 1.47 (± 0.18 SD) times higher in ILC relative to nonneoplastic epithelium, and similar (0.93 ± 0.17 SD) in LCIS and nonneoplastic epithelium (Figure 3). These results indicate that c-Src activity is specifically increased in invasive carcinoma cells compared with both *in situ *lesions (range, 1.25 to 2.07 times) and nonneoplastic epithelium (range, 1.16 to 1.98 times). Similar to the expression pattern of active c-Src, total c-Src was increased in ILC relative to *in situ *lesions and nonmalignant epithelium (see Additional data file 1), suggesting that the elevated c-Src activity is due to increased expression of the kinase.

We further examined whether expression of active c-Src in nonneoplastic epithelium from LBC patients is different from that in normal epithelium from healthy individuals. We performed double immunofluorescence with the clone 28 antibody and an antibody against cytokeratin 18 on epithelial tissue from 10 randomly selected LBC patients and from six breast-reduction surgery patients. For each patient, the ratio between mean c-Src fluorescence intensity and mean cytokeratin 18 fluorescence intensity was calculated. The average fluorescence ratio (c-Src/CK18) was 1.027 (SD, 0.049; range, 0.967 to 1.099) for LBC patients, and 1.086 (SD, 0.114; range, 0.916 to 1.234) for reduction-surgery patients. These results suggest no significant difference exists in c-Src activity between tumor-adjacent and healthy epithelium.

### Increased c-Src activity correlates with the activation of Fak, Stat-3, and the expression of mesenchymal markers in ILC cells

Because c-Src activity was increased in invasive relative to noninvasive cells, the kinase may contribute to the invasiveness of lobular carcinoma cells. To investigate whether c-Src activity in LBC may be associated with an epithelial-mesenchymal transition (EMT), we examined the co-expression of c-Src downstream targets implicated in the transition to an invasive, mesenchymal-like state.

**Figure 4:**
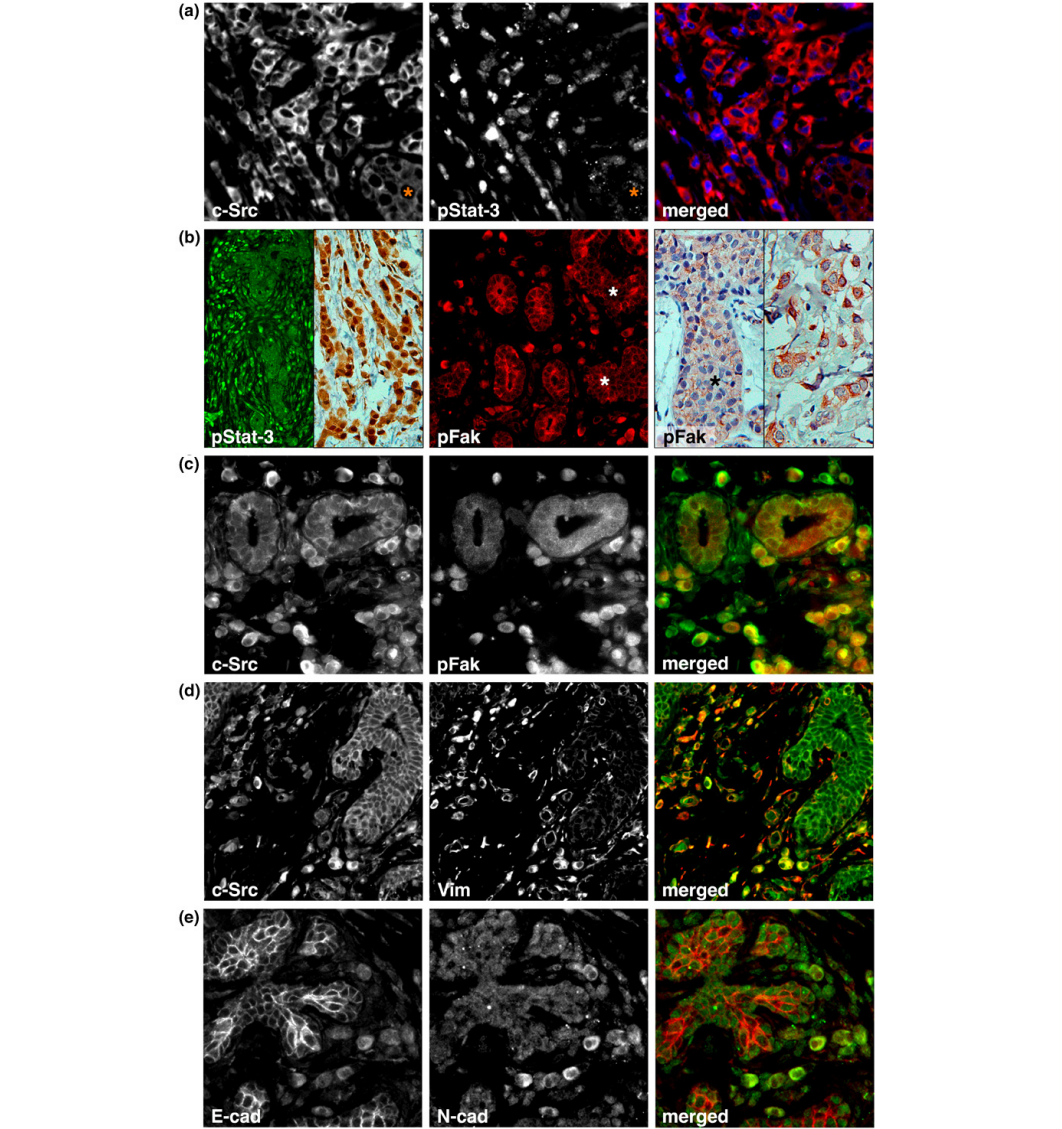
The correlation of c-Src activity with an EMT. **(a) **Nuclear pStat-3 (blue) in invasive lobular carcinoma (ILC) cells with activated c-Src (red). Note the weak nuclear Stat-3 staining in lobular carcinoma *in situ *(LCIS; asterisk). An antibody against total Stat-3 was used as a control. **(b) **Left panel: widespread Stat-3 activation in ILC cells. Middle panel: increased levels of activated Fak in ILC cells surrounding nonneoplastic epithelium and LCIS (asterisks). Right panel: expression of pFak in LCIS (asterisk) and ILC (right). An antibody against total Fak was used as a control. **(c) **Colocalization of active c-Src (green) and active Fak (red) in ILC cells. **(d) **ILC cells with activated c-Src (green) are positive for the mesenchymal marker vimentin (red). **(e) **Expression of the mesenchymal N-cadherin (green) in a subset of invasive cancer cells.

Both Stat-3 and Fak can be activated by c-Src by phosphorylation and are thought to contribute to c-Src-mediated invasion [16,17]. Immunofluorescence using antibodies against the phosphorylated, active forms of Stat-3 and Fak demonstrated that both proteins are active in ILC cells with elevated c-Src activity (Figure 4a to 4c). Nuclear translocation of Stat-3 was barely observed in the *in situ *lesions. In contrast, about 80% of c-Src-positive ILC cells had nuclear expression of activated Stat-3 (Figure 4a and 4b). However, nuclear Stat-3 also was observed in a minority of ILC cells with low levels of active c-Src (data not shown), suggesting that Stat-3 activation may not be strictly dependent on c-Src. Fak activity was low in ALH/LCIS cells (Figure 4b), and elevated in 20% to 40% of ILC cells relative to noninvasive cells. Fak activity in ILC cells strongly correlated with c-Src activity (Figure 4c), suggesting that c-Src is a main activator of Fak in LBC. Further, c-Src-positive ILC cells but not LCIS cells expressed the mesenchymal marker vimentin (Figure 4d). About 20% of ILC cells in addition displayed upregulation of the mesenchymal N-cadherin (Figure 4e).

The increased activation of c-Src and its downstream targets Stat-3 and Fak, together with the expression of mesenchymal markers in ILC cells relative to *in situ *neoplastic cells, associates the c-Src system with the induction of an EMT during progression from *in situ *to invasive disease. Because the majority of ILC cells remain cytokeratin 18 positive, the observed EMT appears incomplete.

### ILC cells with activated c-Src include cells with a high malignant potential

To assess whether increased c-Src activity in ILC cells may be associated with a propensity to metastasize, we examined the expression of proteins thought to mark breast cancer cells with a high malignant potential.

**Figure 5:**
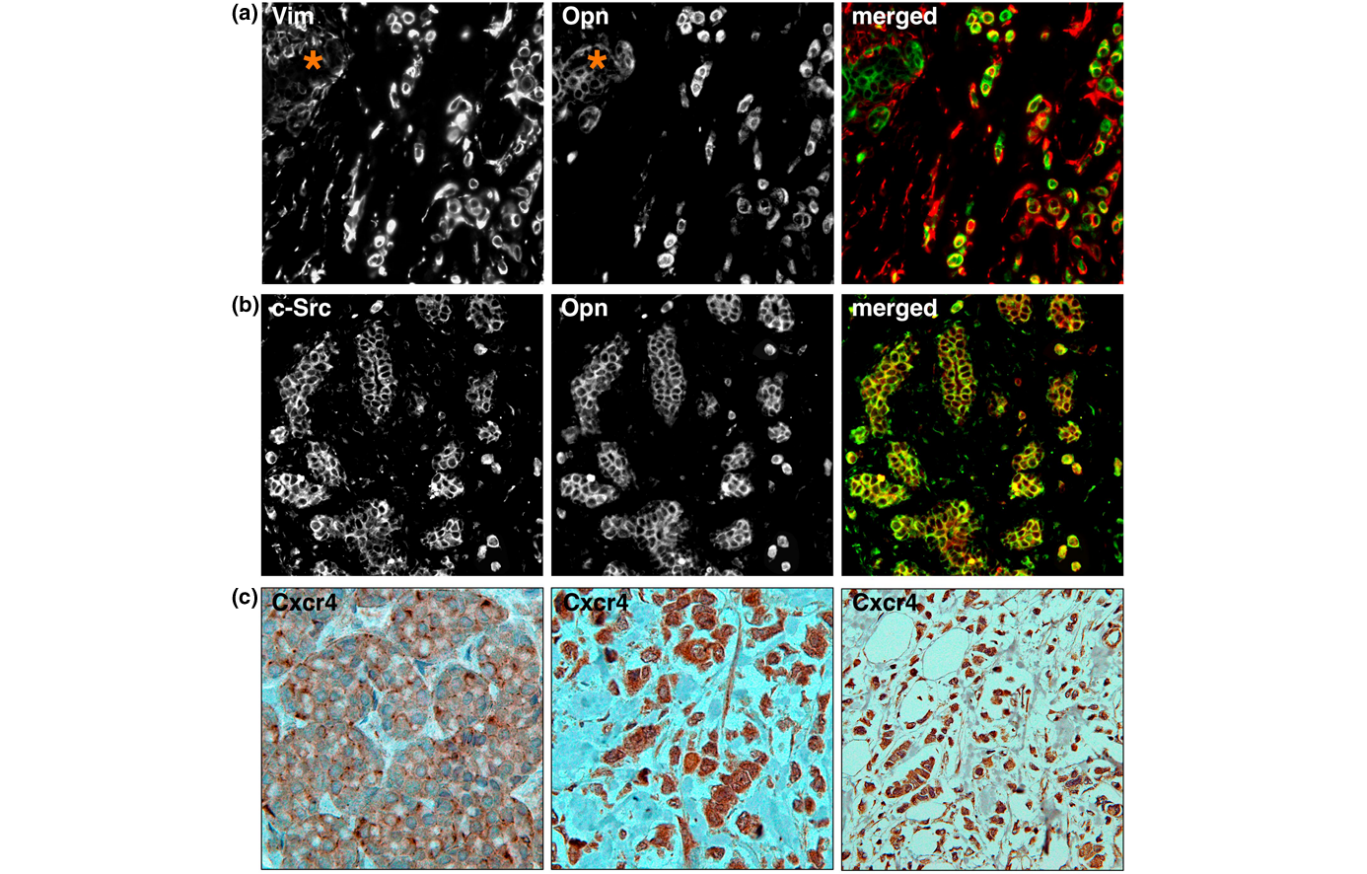
Expression of metastatic markers in invasive lobular breast cancer (LBC) cells. **(a) **Co-expression of osteopontin (green) in invasive lobular carcinoma (ILC) cells positive for vimentin (red). Asterisks mark *in situ *lesions. **(b) **LBC cells with activated c-Src (green) coexpress the bone metastatic marker osteopontin (red). **(c) **Cxcr4, a marker for lymph node metastasis, was weakly expressed in the *in situ *lesions (left) and strongly expressed in the vast majority of invasive cancer cells (middle and right).

Osteopontin (Opn) has been specifically associated with breast cancer metastasis to the bone and appears to be required for this process [18,19]. We observed low to moderate expression of Opn in the *in situ *lesions and strong expression in invasive cancer cells (Figure 5a). Strong Opn expression was present in more than 90% of ILC cells with activated c-Src, consistent with the proposed role of c-Src in the regulation of *OPN *expression [20] and in physiologic bone metabolism [21].

The chemokine receptor Cxcr4 is another protein that has been associated with an unfavorable outcome and the occurrence of lymph node metastasis in breast cancer [22]. We were not able to perform double immunofluorescence for c-Src and Cxcr4 to assess their coexpression, given the reported upregulation of the receptor by c-Src [23]. However, Cxcr4 was strongly expressed in the vast majority of c-Src-positive ILC cells (Figure 5c), suggesting that the increased c-Src activity may be associated with the elevated Cxcr4 levels.

Together, more than 90% of ILC cells displayed increased activation of c-Src and overexpression of both Opn and Cxcr4, suggesting that c-Src activity is associated with an elevated malignant potential of invasive LBC cells.

### ILC cells with activated c-Src include cells positive for the breast cancer stem cell marker Aldh1

**Figure 6:**
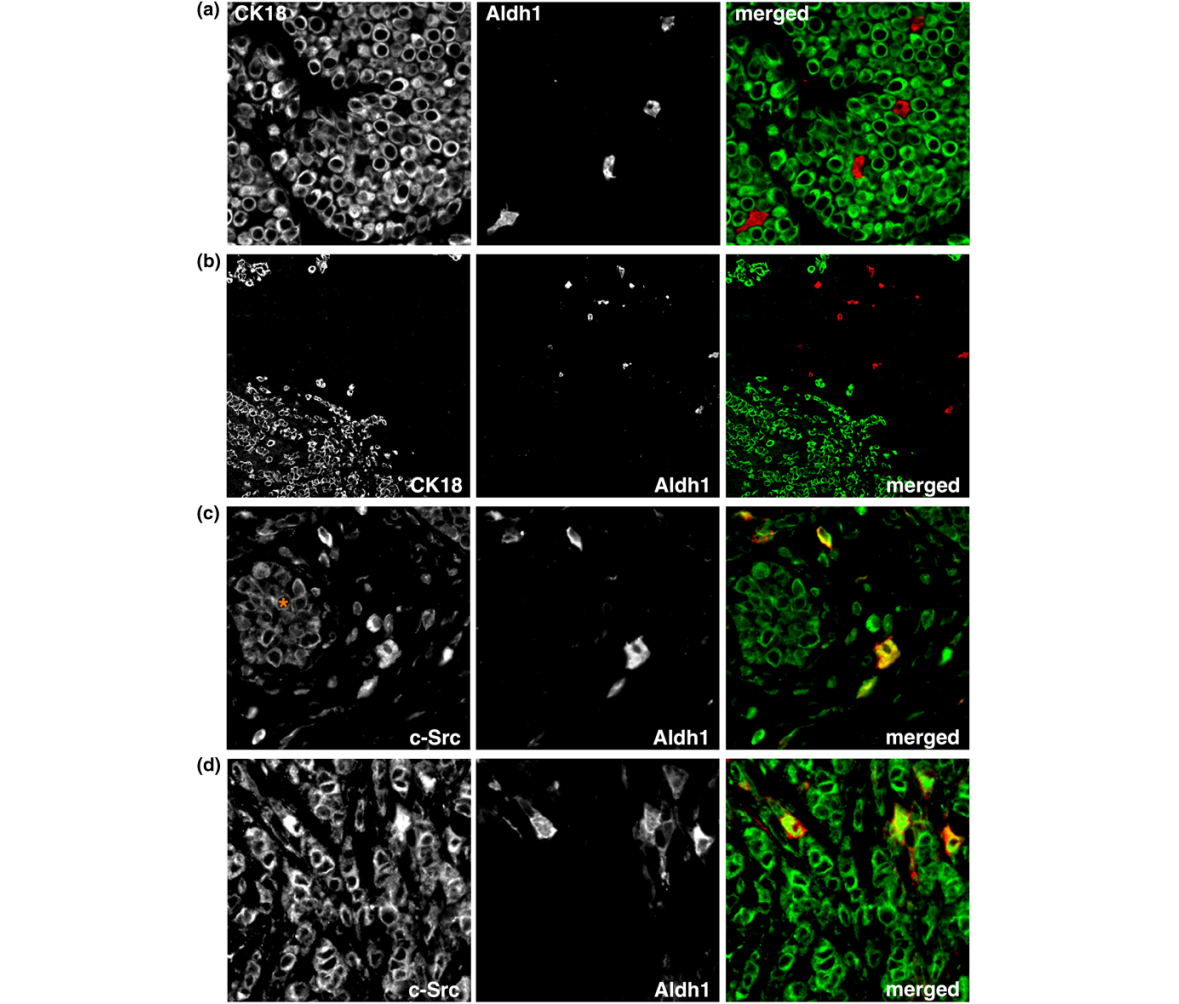
Expression of the breast cancer stem cell marker Aldh1 in lobular breast cancer (LBC). **(a) **A lobular carcinoma *in situ *(LCIS) stained for cytokeratin 18 (green) and aldh1 (red). Note the mutually exclusive expression of the two proteins. **(b) **An invasive lobular carcinoma (ILC) stained for cytokeratin 18 (green) and aldh1 (red). Aldh1-positive cells were frequently observed at invasive fronts. **(c) **Coexpression of aldh1 (red) in ILC cells positive for active c-Src (green). The asterisk marks an LCIS. (d) Aldh1 expression (red) in ILC cells with strong c-Src activity (green).

Breast cancer belongs to the solid tumors believed to be initiated and maintained by cancer stem cells. Originally, these tumor-driving cells were identified as a fraction of breast cancer cells with high and low expression of the cell-surface markers CD44 and CD24, respectively [24]. In our LBC samples, however, CD44^high^/CD24^low ^cells accounted for an average of 60% of all cancer cells and were positive for cytokeratin 18, a marker for differentiated luminal cells (data not shown). We thus examined the expression of Aldh1, another proposed marker for breast cancer stem cells [25]. Indeed, Aldh1 and cytokeratin 18 were expressed in a mutually exclusive way in both LCIS (Figure 6a) and ILC (Figure 6b), consistent with Aldh1 marking undifferentiated breast cancer stem cells. The Aldh1^+ ^cells constituted between 0 and 5% of all neoplastic cells and were found mostly at the invasive front in ILC samples. Notably, essentially all (>95%) Aldh1^+ ^ILC cells also had increased c-Src activity (Figure 6c and 6d). These findings suggest that invasive LBC cells with overactive c-Src include the vast majority of the putative breast cancer stem cells.

### Disease recurrence occurs in LBC patients with a high c-Src kinase activity in their invasive components

**Figure 7:**
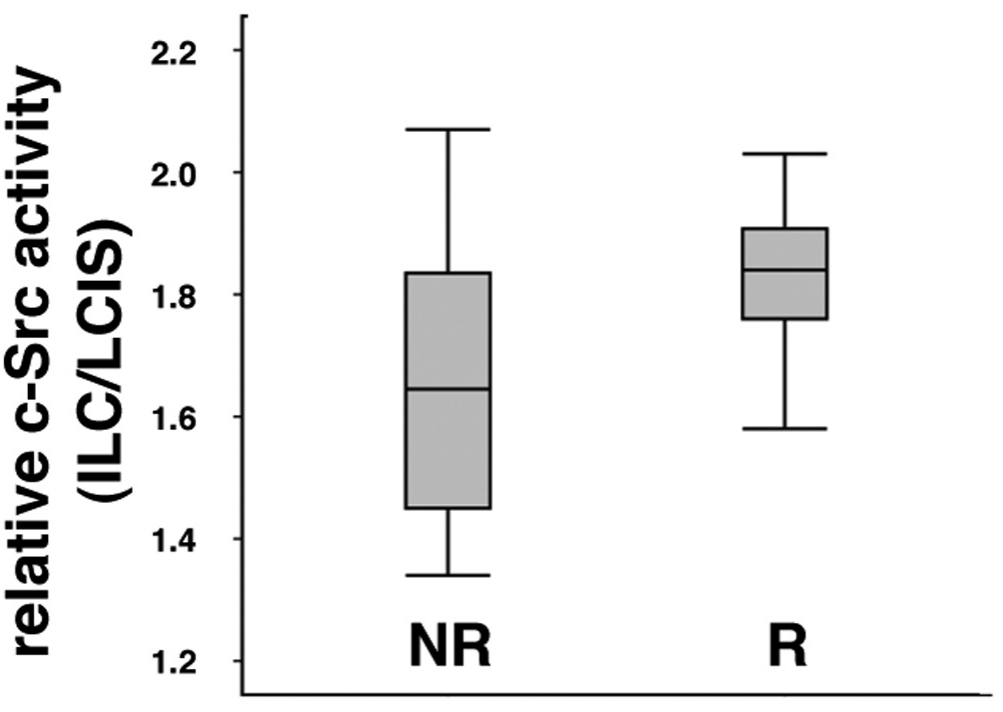
Relative c-Src activity in non-recurrent versus recurrent lobular breast cancer (LBC) patients. The box plot shows the increase in active c-Src fluorescence of invasive lobular carcinomas (ILCs) relative to their adjacent *in situ *lesions in the groups of patients with nonrelapsed (NR) and relapsed (R) disease. The median value is indicated by the lines across the boxes. Box lengths refer to the interquartile range. Lower and upper whiskers represent the minimal and maximal values, respectively.

To assess whether elevated c-Src activity is clinically relevant, we examined an association between disease recurrence and relative c-Src activity. Seven of the 57 LBC patients had relapsed disease. Mean relative c-Src activity (ILC versus LCIS) was 1.82 ± 0.15 SD in the patients with relapse and 1.62 ± 0.24 SD in the patients without relapse (Figure 7). Although this difference was not statistically significant, a tendency was seen for a higher c-Src activity in patients with relapsed compared with nonrelapsed disease (*P *= 0.072; two-tailed Mann-Whitney test). Furthermore, estrogen- and progesterone-receptor status was determined with immunohistochemistry in the seven patients with relapses and in the 10 patients without relapse with the lowest c-Src activity. All examined patients were positive for the hormone receptors (see Additional data file 1), suggesting that the hormone-receptor status was not related to the observed association between c-Src activity and relapse.

The activity of c-Src kinase might therefore be associated with an increased likelihood of recurrence in LBC.

## Discussion

Among the various novel targeted approaches in cancer chemotherapy, inhibition of c-Src kinase appears to hold promise in counteracting invasive stages that ultimately lead to the spread and metastasis of cancer cells. The potential of c-Src to drive invasion-associated mesenchymal changes during carcinoma progression has been well illustrated for colorectal cancer [9]. We recently reported that c-Src may have a similar role in diffuse gastric cancer, a carcinoma morphologically and etiologically related to LBC [10]. Here, we present evidence to extend the invasion-promoting role of c-Src to lobular carcinoma of the breast and to suggest antagonists of the kinase as novel therapeutic options for this delicate disease.

In this study, we observed increased levels of active c-Src in invasive LBC cells relative to adjacent *in situ *LBC lesions or to nonneoplastic epithelia in the majority of LBC patients. We used immunofluorescence intensity from an antibody against activated c-Src as a measure for c-Src activity. Such an approach can provide only a rough estimate for c-Src activity, as the actual kinase activity will depend on more parameters than the expression level alone. Activity assays in fresh tissue samples would be required for a clean assessment; however, given that early-stage LBC is an incidental microscopic finding in most cases, tissue availability is usually limited to archived specimens. Nonetheless, the consistently increased levels in ILC relative to LCIS/ALH and epithelium suggest that a biologic function underlies the expression pattern of activated c-Src.

Indeed, the potential downstream targets of c-Src, Stat-3 and Fak, were both activated in ILC but not in LCIS/ALH or nonneoplastic epithelium, consistent with an actual increase in c-Src activity. Stat-3 is a well-defined c-Src target, in invasive breast cancer cells and animal models, and is required for experimental breast cancer metastasis [17,26,27]; in addition, its activity correlates with that of c-Src in advanced breast cancer [28]. Even though Stat-3-mediated feedback downregulation of c-Src has been reported [17], it is highly likely that the specific activation of Stat-3 is due to the elevated Src activity in ILC cells. Conversely, extensive cross-regulation exists between c-Src and Fak, and mutual control has been observed in breast cancer cells for the two kinases [29-32]. However, because ILC cells with active Fak were contained as a subpopulation within the c-Src-positive ILC cells, it is more likely that c-Src was upstream in our studied cases. We thus propose that the elevated c-Src activity contributes to the activation of Stat-3 and Fak in ILC cells.

Because the increased expression of active c-Src and the activation of its downstream targets was paralleled by the expression of mesenchymal markers, we further propose that an EMT is involved in the acquisition of invasiveness of LBC cells. c-Src, Stat-3, and Fak have all been previously associated with the induction/execution of EMTs; however, whether an EMT plays a part in LBC progression is not established. Experimental models of lobular carcinoma clearly support this hypothesis [33], but evidence from human tissue is less straightforward. Notably, recent studies on clinical material indicate strong expression of EMT-associated genes in invasive LBC [34,35]; however, the majority of ILC cells remain cytokeratin positive, arguing against a mesenchymal phenotype. This apparent conflict is easily resolved if one accepts that EMTs in a pathologic context do not need to be complete. The simultaneous expression of epithelial and mesenchymal markers strongly suggests that epithelial ILC cells have gained some mesenchymal features that facilitate invasion. To this end, a partial EMT is consistent with a functional acquisition of novel properties needed at a specific disease stage rather than a proper execution of an intrinsic developmental program. A partial EMT also would be consistent with the characteristic feature of LBC, downregulation of E-cadherin, as loss of adhesion is one key step during the process of an EMT. A partial EMT may therefore suffice to turn stationary but nonadhesive LCIS cells into migratory ILC cells. Of note, mesenchymal features were evident only in ILC but not in LCIS. This observation indicates that downregulation of E-cadherin alone is not enough to induce an EMT *in vivo*, contrary to the perception of E-cadherin as a master regulator of EMTs [36,37], but supported by the observation that forced expression of E-cadherin cannot reverse the EMT-induced, mesenchymal phenotype in experimental breast cancer [33,34].

What leads to the relative upregulation of activated c-Src in ILC cells is not known but appears to be related to an increased protein amount. Amplification of the gene encoding c-Src has not been reported; however, gains at 20q11-13 (encompassing the *SRC *locus at 20q12-13) have repeatedly been observed in ILC [38-42]. Therefore, amplification of *SRC *might be a mechanism to account for the increased c-Src activity. An alternative pathway recently was demonstrated for invasive ductal carcinoma cells, in which upregulation of the homeobox transcription factor Msx2 causes activation of c-Src and a concomitant EMT [43]. Furthermore, the very high proportion of ILC with active c-Src could suggest that increased c-Src activity might be a consequence of E-cadherin downregulation itself. Consistent with this notion, E-cadherin degradation by Ca^2+ ^depletion has been shown to lead to c-Src activation in breast cancer cells [44]. Conversely, functional E-cadherin adhesion also can lead to c-Src activation, with c-Src either reinforcing or weakening the adhesive contacts [45]. This complex interplay might explain why c-Src activity and invasiveness appear to increase only slowly after E-cadherin downregulation.

Whatever the mechanism, our data indicate that inhibition of c-Src activity would affect ILC cells that appear to have a high malignant potential. More than 90% of ILC cells displayed increased c-Src activity and were positive for the metastatic markers Opn and Cxcr4. In addition, the ILC cells with increased c-Src activity also included most of the cells positive for the breast cancer stem cell marker Aldh1. It is not clear whether c-Src inhibition would reverse the Opn/Cxcr4/Aldh1-positive phenotype. If not, c-Src antagonists may simply reduce the spread of highly malignant ILC cells. However, both the *OPN *and *CXCR4 *genes can be induced by c-Src [20,23], and the induction of an EMT has been associated with the generation of breast cancer stem cells [46]. It thus remains possible that inhibition of c-Src could result in the abrogation of an EMT along with a reduced number of Aldh1-positive cells and the suppression of Opn and Cxcr4 production. In such a case, c-Src antagonists may effectively reduce the spread and the malignant potential of ILC cells. Some support for a clinical benefit for c-Src antagonists in LBC comes from our observation that patients with relapses tended to have high levels of active c-Src compared with patients without relapses. A larger patient cohort will be needed to determine whether high c-Src activity is indeed associated with a higher likelihood of relapse in LBC. We also were not able to perform a Kaplan-Meier survival analysis, as the follow-up times of our patients were too heterogeneous. Notwithstanding these limitations, the observed tendency is consistent with the association of high c-Src activity with lower recurrence-free survival in ductal breast cancer patients [6]. Together, it appears likely that inhibitors of c-Src kinase will interfere with the spread of malignant LBC cells and affect the outcome of patients with lobular carcinoma.

## Conclusions

In this study, we observed increased expression levels of active c-Src in ILC relative to LCIS and nonneoplastic epithelia from LBC patients. The increase in active c-Src was paralleled by the activation of EMT-associated c-Src downstream targets and the expression of mesenchymal markers. These findings provide *in vivo *evidence for a contribution of c-Src kinase to the progression of lobular carcinoma to invasive disease and suggest that c-Src promotes LBC invasiveness by the acquisition of mesenchymal features. ILC cells with active c-Src further expressed markers of metastatic breast cancer and included presumed breast cancer stem cells positive for Aldh1. Together with the observation of high c-Src levels in patients with relapses, our data suggest a clinical benefit of c-Src inhibition in LBC patients, advocating the evaluation of c-Src inhibitors as novel chemotherapeutic options in lobular carcinoma.

## Abbreviations

ALH: atypical lobular hyperplasia; EMT: epithelial-mesenchymal transition; FFPE: formalin-fixed paraffin-embedded; ILC: invasive lobular carcinoma; LBC: lobular breast cancer; LCIS: lobular carcinoma *in situ*.

## Competing interests

The authors declare that they have no competing interests.

## Authors' contributions

DZ performed the immunochemistry experiments, measured c-Src immunofluorescence, and helped to write the paper and draft the figures. HSY confirmed the pathology, selected the tissue, helped to design the study, and performed E-cadherin immunochemistry. AA performed the statistical analyses and helped to draft and write the manuscript. DP collected the clinical data and helped to draft the manuscript. RF helped with the immunochemistry and confirmed the pathology. PG helped to design the study and write the manuscript. BH conceived the study, wrote the manuscript, and prepared the figures. All authors read and approved the final manuscript.

## Supplementary Material

Additional file 1 A figure showing the expression levels of total (active and inactive) c-Src kinase in LBC lesions and the estrogen/progesterone receptor status of patients with relapsed versus unrelapsed LBC. Click here for file
