# Supplementary figures and images for: Increased levels of active c-Src distinguish invasive from in situ lobular lesions
Source: Breast Cancer Res. 2009 Jul 7;11(4):R45. doi: 10.1186/bcr2332 (PMC2750104; doi:10.1186/bcr2332)

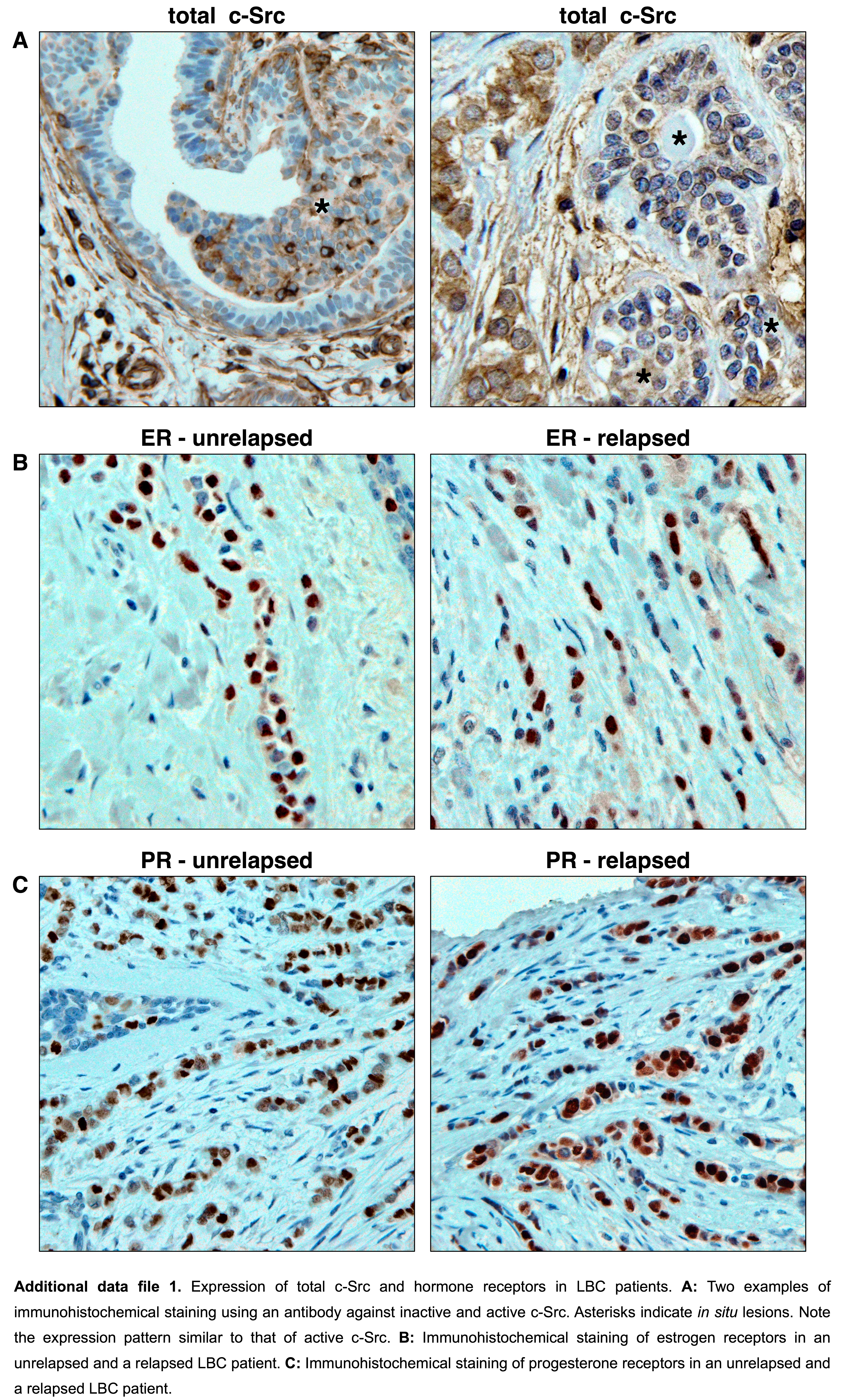

Supplement: Additional file 1 — A figure showing the expression levels of total (active and inactive) c-Src kinase in LBC lesions and the estrogen/progesterone receptor status of patients with relapsed versus unrelapsed LBC. [file bcr2332-S1.jpeg]
